# A data-driven approach to categorizing early life adversity exposure in the ABCD Study

**DOI:** 10.1186/s12874-023-01983-9

**Published:** 2023-07-07

**Authors:** Natalia Orendain, Ariana Anderson, Adriana Galván, Susan Bookheimer, Paul J. Chung

**Affiliations:** 1 Center for Cognitive Neuroscience, University of California, Los Angeles, CA USA; 2 Department of Psychiatry and Biobehavioral Sciences, University of California, Los Angeles, CA USA; 3 David Geffen School of Medicine, Semel Institute for Neuroscience and Human Behavior, University of California, Los Angeles, CA USA; 4 Department of Psychology, University of California, Los Angeles, CA USA; 5 Departments of Pediatrics and Health Policy & Management, University of California, Los Angeles, CA USA; 6 Department of Health Systems Science, Kaiser Permanente Bernard J. Tyson School of Medicine, Pasadena, CA USA

**Keywords:** ACEs, Early life adversity exposure, Problematic behaviors, Factor analysis, CBCL

## Abstract

**Background:**

Adversity occurring during development is associated with detrimental health and quality of life outcomes, not just following exposure but throughout the lifespan. Despite increased research, there exists both overlapping and distinct definitions of early life adversity exposure captured by over 30 different empirically validated tools. A data-driven approach to defining and cataloging exposure is needed to better understand associated outcomes and advance the field.

**Methods:**

We utilized baseline data on 11,566 youth enrolled in the ABCD Study to catalog youth and caregiver-reported early life adversity exposure captured across 14 different measures. We employed an exploratory factor analysis to identify the factor domains of early life adversity exposure and conducted a series of regression analyses to examine its association with problematic behavioral outcomes.

**Results:**

The exploratory factor analysis yielded a 6-factor solution corresponding to the following distinct domains: 1) physical and sexual violence; 2) parental psychopathology; 3) neighborhood threat; 4) prenatal substance exposure; 5) scarcity; and 6) household dysfunction. The prevalence of exposure among 9-and 10-year-old youth was largely driven by the incidence of parental psychopathology. Sociodemographic characteristics significantly differed between youth with adversity exposure and controls, depicting a higher incidence of exposure among racial and ethnic minoritized youth, and among those identifying with low socioeconomic status. Adversity exposure was significantly associated with greater problematic behaviors and largely driven by the incidence of parental psychopathology, household dysfunction and neighborhood threat. Certain types of early life adversity exposure were more significantly associated with internalizing as opposed to externalizing problematic behaviors.

**Conclusions:**

We recommend a data-driven approach to define and catalog early life adversity exposure and suggest the incorporation of more versus less data to capture the nuances of exposure, e.g., type, age of onset, frequency, duration. The broad categorizations of early life adversity exposure into two domains, such as abuse and neglect, or threat and deprivation, fail to account for the routine co-occurrence of exposures and the duality of some forms of adversity. The development and use of a data-driven definition of early life adversity exposure is a crucial step to lessening barriers to evidence-based treatments and interventions for youth.

**Supplementary Information:**

The online version contains supplementary material available at 10.1186/s12874-023-01983-9.

## Background

Adversity occurring during development is associated with a host of detrimental health and quality of life outcomes, not just following exposure but throughout the lifespan [1, 2]. In addition to a dose–response relationship with risk for morbidity and premature mortality [3, 4], early life adversity (ELA) is associated with a higher incidence of problematic behaviors, neural alterations and psychopathology risk [5]. ELA research is increasingly addressing the nuances of exposure—type, age of onset, frequency, duration, and relationship with the perpetrator—to better understand the physiological mechanisms associated with outcomes risk and aptitude for resilience [1, 6]. Despite increased research on ELA and its association with certain sociodemographic features, such as low socioeconomic status (SES) [4], inconsistent physiological and behavioral results and lack of replicability [5, 7, 8] muddle findings and create barriers to evidence-based treatments and interventions. A data-driven approach utilizing a population-based sample with a breadth of ELA exposures is crucial to define and catalog exposure, better understand associated outcomes and advance the field.

Both across and within disciplines, including basic science, psychology, neuroimaging, epidemiology, education and policy, there exists an overlapping yet distinct range of ELA definitions. Even the term ELA is used inconsistently with early life stress, childhood maltreatment, and adverse childhood experiences or ACEs [8, 9]. In this study, our use of ELA refers to any adversity or trauma occurring during development and thus includes early life stress, childhood maltreatment and ACEs. Traditionally, development has referred to birth to 18 years of age, sometimes including prenatal development [9]; however, this definition does not address the neurodevelopmental processes that continue to unfold past age 18 and throughout one’s twenties. While ACEs, as a term, contains a specific set of exposures and gained traction with the landmark CDC-Kaiser Study under the same name [3], over 30 different tools [8] have been used to empirically study adversity during development, e.g., Childhood Trauma Questionnaire [10], Child Abuse and Trauma Scale [11] and the Maltreatment and Abuse Chronology of Exposure (MACE) scale [12]. Scientists and clinicians have also suggested broad categorizations of adversity exposure to help explain disparities in physiological findings. Categorizations include the broad domains of abuse and neglect [13], active and passive adversity [14], and threat and deprivation [15]. 

Lack of replicability and disparate physiological and behavioral findings may in part be attributable to methodological differences across studies. A recent meta-analysis found significant differences in findings attributable to ELA exposure when obtained prospectively instead of retrospectively; the vast majority of ELA studies fall under the latter [16]. Given the prevalence of ELA—62% of adults have experienced at least one ELA, and 25% have experienced 3 or more [4]—and its acute and long-term correlates with overall health and well-being, there is a need for a systematic data-driven approach to measure and categorize adversity exposure in youth. Such an approach could aid in establishing a consistent manner with which to define and measure ELA, improve study reproducibility, and elucidate inconsistences in findings.

The current study aims to better understand the structure of ELA exposure among 9- and 10-year-old youth, and to further examine its relationship with behavioral outcomes. As there is not a single questionnaire nor gold standard by which to measure ELA exposure, the Adolescent Brain Cognitive Development (ABCD) Study incorporates adversity-related questions from a variety of questionnaires, given to both the youth and the caregiver. The ABCD Study is a 10-year longitudinal study of youth development. We first performed an exploratory factor analysis (EFA) on 11,566 nine- and ten-year-old youth enrolled in the ABCD Study at baseline, using both youth and caregiver-reported questions from 14 different measures. The adversity measures capture exposure prenatally to the youth’s current age of nine or ten years and are predominately caregiver reported. We hypothesized that adversity domains derived from the EFA would overall align with and complement the domains established by the CDC-Kaiser ACE’s Study given that the original categorizations of exposure were broad yet discrete in nature. Specifically, we hypothesize distinct domains of abuse, neglect, household dysfunction, in addition to neighborhood threat and violence, which is not included in the CDC-Kaiser ACE’s Study. Within abuse, we hypothesize distinct domains of physical and sexual abuse, but do not hypothesize emotional abuse to be distinctly identified due to the age of the sample and their developing ability to name and decipher their emotional well-being. Similarly, within neglect, we do not hypothesize distinct categories of emotional and physical neglect, again due to the age of the sample. Within household dysfunction, we hypothesize the subdomains of parental psychopathology and mother treated violently. Additionally, we hypothesized that higher scores on the distinct factors would correlate with greater problematic behaviors in comparison to youth without any ELA exposure. To examine the relationship between ELA subtypes and problematic behaviors, a series of linear and logistic regression analyses were utilized.

## Methods

### Protocol

The present study used the National Data Archive, ABCD version 2.01 baseline data set collected between 2016 and 2018 from the ABCD study, the largest longitudinal neuroimaging study of youth development. Over 10,000 youth from 21 different research sites in the United States are enrolled in this 10-year longitudinal study [17]. Procedures, sampling and recruitment [17–19] for the ABCD study have been described previously. Caregivers provided written informed consent and children provided assent for participation in the study. All procedures were approved by a central institutional review board, and each site has a detailed protocol in place to address reported adversity exposure. The University of California, Los Angeles, institutional review board has indicated that analyses using the publicly released ABCD Study data are not human subjects research and therefore do not require their own approval.

### Measures

#### Sociodemographic characteristics

A caregiver-completed demographic questionnaire was used to gather information regarding youth’s age, sex, race and ethnicity, as well as family income and primary caregiver’s education. These demographic features were employed as covariates in subsequent analyses.

#### Early life adversity exposure

Early life adversity was measured through a series of 14 questionnaires, assessing exposure throughout the lifespan among 9- and 10-year-olds. Across the questionnaires, 47 variables were identified that captured different forms of adversity exposure, including: physical, sexual and emotional abuse; emotional and physical neglect; loss of parent; domestic violence; parental psychopathology and drug use; and threatening experiences (e.g., witnessing community violence, experiencing death threats). Due to the sensitive nature of the questions and the age of the youth, most of the adversity variables were parent-reported. Youth-report was used to measure household dysfunction and parental emotional abuse. All adversity variables were binarized to indicate the presence or absence of exposure. In addition to the factor loading score, a raw count of exposure was derived from the six domains. The raw count of exposure within each domain, not the factor score, was normalized given that the six domains were comprised of a different number of corresponding exposure questions. For example, the first factor loading was comprised of 9 questions, the second loading was comprised of 8 questions, and the third loading was comprised of 3 questions. To ensure that one domain didn’t carry more or less weight than the other domains due to the number of questions assessing exposure within the domain, the raw count of ELA exposure by domain was standardized across questions and questionnaires. The raw count was reported in the descriptive tables and the factor scores were utilized in the regression models.

#### Clinical outcomes

The parent-reported Child Behavior Checklist (CBCL) [20] was used to assess children’s internalizing problems, externalizing problems, and total problems, the latter encompassing the sum of all internalizing and externalizing behaviors. Internalizing problematic behaviors can be assessed by anxious, depressed, and withdrawn behaviors, while externalizing behaviors include rule breaking and aggressive behavior. This measure captured problems over the prior 6 months and raw scores were converted to t scores, with a t score less than 60 representing normal functioning [20].

### Statistical analyses

#### Overview

All data were analyzed using R version 3.5.1 [20]. Sociodemographic characteristics and clinical outcomes were inspected for normality by examining skewness and kurtosis. Youth with an adversity score of zero across all domains, such that no form of adversity exposure was endorsed or captured, served as the study’s control group (*n* = 915). Chi-squared and independent t-tests were performed to examine differences in demographic characteristics across youth with early life adversity exposure and controls (see Table 1). To adjust for multiple comparisons across all analyses, we utilized Benjamini–Hochberg corrections at *p* < 0.05.

**Table 1:** Demographic characteristics of ABCD study youth

	No. (%)		
Characteristic	**Total (*n* = 11,566)**	**Adversity Exposed (*n* = 10,651)**	**Controls (*n* = 915)**
**Age in Years (M, SD)**	9.47 (0.50)	9.47 (0.50)	9.52 (0.50)
**Sex**			
Male	6050 (52.7)	5611 (52.7)	439 (48.0)
Female	5517 (47.3)	5042 (47.3)	476 (52.0)
**Race and Ethnicity**			
White	6018 (52.0)	5623 (52.8)	395 (43.2)
Black	1733 (15.0)	1581 (14.8)	152 (16.6)
Asian	2341 (20.2)	2106 (19.8)	235 (25.7)
Other	244 (2.1)	183 (1.7)	61 (6.7)
Hispanic	1230 (10.6)	1158 (10.9)	72 (7.9)
**Household Income**			
0–24,999	1596 (13.8)	1471 (13.8)	125 (13.7)
25,000–49,999	1545 (13.4)	1446 (13.6)	99 (10.8)
50,000–74,999	1456 (12.6)	1360 (12.8)	96 (10.5)
75,000–99,999	2524 (21.8)	2303 (21.6)	221 (24.2)
100,000 +	4445 (38.4)	4071 (38.2)	374 (40.9)
**Primary Caregiver's Educational Attainment**			
Less Than HS Diploma	766 (6.6)	708 (6.6)	58 (6.3)
HS Diploma/GED	1232 (10.7)	1132 (10.6)	100 (10.9)
Some College or AA Degree	3401 (29.4)	3183 (29.9)	218 (23.8)
Bachelors Degree	3255 (28.1)	2974 (27.9)	281 (30.7)
Graduate and Professional School	2912 (25.2)	2654 (24.9)	258 (28.2)
**Clinical Outcomes, t-score ≥ 60 (M, SD)**			
Internalizing Problems	1940 (16.7)	1880 (17.7)	60 (6.6)
Externalizing Problems	1211 (10.5)	1190 (11.1)	21 (2.3)
Total Problems	1414 (12.2)	1389 (13.0)	25 (2.7)
**Adversity Exposure**			
Physical and Sexual Violence	843 (7.3)	843 (7.9)	-
Parental Psychopathology	9413 (81.4)	9413 (88.4)	-
Neighborhood Threat	2304 (19.9)	2304 (21.6)	-
Prenatal Substance Exposure	1224 (10.6)	1224 (11.5)	-
Scarcity	1348 (11.7)	1348 (12.7)	-
Household Dysfunction	4867 (42.1)	4867 (45.7)	-

#### Exploratory factor analysis

The 47 variables from the 14 surveys were first binarized to account for the presence or absence of measurement of interest, such as abuse frequency. This rescaled all items across all instruments for further analyses. To organize, categorize and weigh the study’s adversity variables, we utilized the domains derived from the exploratory factor analysis due to its noted ability to capture latent constructs [21]. We utilized the “fa” function in the psych package to perform our factor analysis in R [22]. To determine the rotation type employed in our factor analysis, we assessed the correlations among factors using an oblique rotation [23]. Factor correlations were strongly driven by the data and therefore an oblique rotation (promax in R) was kept. We examined the matrices for singularity and multicollinearity and utilized Bartlett’s test of sphericity and the Kaiser–Meyer sampling adequacy to ensure that the assumptions for an exploratory factor analysis were not violated. The number of factors, 6, was selected using the “Eigenvalue greater-than-1-rule” in conjunction with examining the scree plot, with agreement between the two methods. Parallel analyses showed no difference with fit among principal factor solution, minimum residual, and generalized weighted least squares. Individual variables were considered to load on a given factor if the factor loading was ≥ 0.40. For items with loadings on two or more factors, analyses were repeated until all items strongly loaded on a single factor. Cross-loading items smaller than our factor loading threshold of 0.40 were removed. Using the factors from the final analysis with the entire sample, factor scores were calculated for each youth within each of the six domains.

#### Regression modeling: relationship with CBCL outcomes

To understand the relationship between the six factor domains and three CBCL outcomes, regression models utilizing the CBCL behavioral outcomes were performed while controlling for common covariates, including age, sex, race and ethnicity of youth, primary caregiver’s education and family income. A set of 3 linear regression models were run on internalizing, externalizing and total problematic behaviors as continuous outcomes, including clinically and non-clinically significant values. A set of 3 additional binomial logistic regression models were run on binarized internalizing, externalizing and total problematic behaviors, such that a response of 1 corresponded with a clinically-significant CBCL score of 60 and above; a response of zero corresponds with non-clinically significant normal functioning. Corresponding metrics, including odds ratios for binomial regression models, of these regression models are presented in the later tables. 

## Results

### Overview

The prevalence of at least one form of ELA was 92.1% among our sample of 11,566 9- and 10-year-olds across the following domains: 1) physical and sexual violence; 2) parental psychopathology; 3) neighborhood threat; 4) prenatal substance exposure; 5) scarcity; and 6) household dysfunction. 81.4% of youth were exposed to parental psychopathology, 42.1% reported household dysfunction, 19.9% experienced neighborhood threat, 11.7% faced scarcity, 10.6% were exposed to prenatal substance use, and 7.3% reported physical and sexual violence exposure. Youth with ELA and controls were statistically different from one another across the following sociodemographic characteristics: sex (χ^2^ = 7.44; *p* = 0.006), race and ethnicity (χ^2^ = 136.25; *p* < 0.0001), family income (χ^2^ = 16.64; *p* = 0.002) and primary caregiver’s education (χ^2^ = 12.29; *p* = 0.015) (see Table 1). Additionally, youth with ELA endorsed more internalizing, externalizing and total problematic behaviors (χ^2^(5, *N* = 11,566) = 8.84, *p* = 0.012).

### Exploratory factor analysis

No evidence for singularity and multicollinearity was found for the correlation matrix. Evaluation of the correlation matrix showed a correlation of the items between -0.089 and 0.691.

The determinant of the matrix was not equal to the identity matrix (< 0.00001). Bartlett’s test of sphericity was significant (χ^2^(1176,*N* = 11,566) = 80,020.79, *p* < 0.01), suggesting that there was enough variability in the items to perform the factor analysis. The Kaiser Meyer Olkin measure of sampling adequacy was acceptable at 0.80 and indicated common variance among the items.

Out of the 47 variables included in the factor analysis, 30 variables loaded on six unique domains. A six-factor solution was identified for the final factor analysis utilizing a principal factor solution and oblique rotation. The eigenvalue for the first six factors ranged from 1.02 to 3.45 and explained 22.4% of the variation in this construct. All other eigenvalues were less than 1 and accounted for less than 10% of the variation. Our selected model’s fit corresponds to: root mean square error of approximation (RMSEA) value = 0.02; and Tucker-Lewis index (TFI) and comparative fit index (CFI) values > 0.85. No variables loaded on more than one factor. ﻿As shown in Table 2, this solution gives clearly interpretable factors entitled: 1) physical and sexual violence; 2) parental psychopathology; 3) neighborhood threat; 4) prenatal substance exposure; 5) scarcity; and 6) household dysfunction.

**Table 2:** Early life adversity factor structure (loadings) in 9- and 10-year-olds at baseline (*n* = 11,566)

	Rotated factor-pattern (standardized regression coefficients)					
Lifetime exposure	**Factor 1: Physical and Sexual Violence**	**Factor 2: Parental Psycho-pathology**	**Factor 3: Neighbor- hood Threat**	**Factor 4: Prenatal Substance Exposure**	**Factor 5: Scarcity**	**Factor 6: Household Dysfunction**
Beaten by family member	0.797					
Beaten by non-family member	0.795					
Received bruises from beating	0.575					
Sexually assaulted by family member	0.653					
Sexually assaulted by non-family member	0.571					
Sexually assaulted by peer	0.390					
Witnessed community shooting/stabbing	0.492					
Threatened to be killed by family member	0.545					
Threatened to be killed by non-family member	0.593					
Parental alcohol misuse		0.463				
Parental drug misuse		0.478				
Parental depression		0.605				
Parental bipolar disorder		0.437				
Parental psychosis		0.377				
Parent sought mental health counseling		0.630				
Parent hospitalized for mental health		0.657				
Parent attempted/committed suicide		0.501				
Neighborhood safety			0.769			
Neighborhood violence			0.796			
Prenatal tobacco exposure				0.344		
Prenatal alcohol exposure				0.388		
Prenatal cannabis exposure				0.422		
Prenatal crack/cocaine exposure				0.574		
Prenatal heroin/morphine exposure				0.402		
Prenatal opioid exposure				0.459		
Food insecurity					0.679	
Utility services (gas, electric) turned off					0.691	
Family members hit one another						0.495
Family members fight						0.615
Family members criticize						0.462

Given the prevalence of parental psychopathology, a closer examination was conducted demonstrating that the greatest weight comes from the following three sub-types of parental psychopathology exposure: parental hospitalization due to mental health concerns (factor loading: 0.657; prevalence: 36.7%), parent utilization of mental health counseling due to mental health concerns (factor loading: 0.630; prevalence: 70.3%), and parental depression (factor loading: 0.605; prevalence: 61.3%). Additionally, there is a moderate correlation between parental hospitalization and parental utilization of mental health counseling (*r*(10,649) = 0.34, *p* < 0.0001). These questions were completed by the youth’s primary caregiver in regard to the youth’s biological parent; the two of which were not always the same.

### Regression modeling: relationship with CBCL outcomes

The presence of clinically significant internalizing behaviors was reported in 17.7% of youth with at least one form of adversity exposure as opposed to 6.6% of controls (χ^2^(1,*N* = 11,566) = 74.28, *p* < 0.0001); clinically significant externalizing behaviors were reported in 11.1% of youth with at least one form of adversity exposure as opposed to 2.3% of controls (χ^2^(1,*N* = 11,566) = 70.84, *p* < 0.0001); total clinically significant problematic behaviors were evident in 13.0% of ELA youth versus 2.7% among controls (χ^2^(1,*N* = 11,566) = 83.45, *p* < 0.0001).

Youth with higher factor scores across the following domains had more internalizing problems: physical and sexual violence, parental psychopathology, and scarcity. Conversely, individuals with higher factor scores across the following domains had higher externalizing problems: neighborhood threat, prenatal substance exposure, and household dysfunction (see Table 3).

**Table 3:** Linear associations between factor score domains and clinical outcomes (*n* = 11,566)

	Standardized Regression Coefficient (SE)					
Lifetime exposure	**Internalizing Problems**	***p*-value**	**Externalizing Problems**	***p*-value**	**Total Problems**	***p*-value**
Factor 1: Physical and sexual violence	0.61 (0.11)	** < .0001**	0.50 (0.10)	**.0001**	0.68 (0.11)	**.0001**
Factor 2: Parental psychopathology	2.76 (0.12)	** < .0001**	2.42 (0.11)	**.0001**	3.26 (0.12)	**.0001**
Factor 3: Neighborhood threat	1.34 (0.13)	** < .0001**	1.40 (0.13)	**.0001**	1.65 (0.14)	**.0001**
Factor 4: Prenatal substance exposure	1.00 (0.13)	** < .0001**	1.68 (0.13)	**.0001**	1.70 (0.14)	**.0001**
Factor 5: Scarcity	0.37 (0.17)	.035	0.32 (0.17)	.058	0.43 (0.18)	.019
Factor 6: Household dysfunction	1.44 (0.14)	** < .0001**	2.48 (0.13)	**.0001**	2.45 (0.14)	**.0001**

Linear regression analyses controlled for age, sex, race/ethnicity of youth, primary caregiver's education and family income

*SE* standard error

While controlling for age, sex, race/ethnicity of the youth, and family income, all forms of adversity exposure except for scarcity were significantly associated with greater internalizing, externalizing and total problematic behaviors (*p* < 0.0001) (see Tables 4, 5 and 6). In particular, parental psychopathology, household dysfunction and neighborhood threat demonstrated the greatest association with problematic behaviors, while controlling for age, sex, race, ethnicity and family income. A cumulative adversity exposure score was calculated for each youth, created by summing the adversity scores across the six domains. The relationship between cumulative adversity exposure and problematic behaviors is included in Table 6.

**Table 4:** Linear regression of early life adversity and CBCL symptomology

	*Internalizing Behaviors*			
	B	SE	*t* value	*P*-value
Physical and Sexual Violence	0.45	0.10	4.47	< .0001
Parental Psychopathology	2.54	0.11	22.52	< .0001
Neighborhood Threat	0.95	0.12	8.06	< .0001
Prenatal Substance Exposure	0.42	0.12	3.43	.0006
Scarcity	0.3	0.12	2.56	.0106
Household Dysfunction	0.96	0.13	7.55	< .0001
Age	0.42	0.19	2.23	.026
Sex: Male	-1.76	0.19	-9.29	< .0001
Race and Ethnicity: White				
Black	-3.04	0.32	-9.65	< .0001
Asian	0.14	0.27	0.53	.5981
Other	-0.99	0.67	-1.49	.1365
Hispanic	-0.04	0.32	-0.13	.8937
Family Income: 0–24,999				
25,000–49,999	-0.43	0.37	1.18	.2400
50,000–74,999	-0.71	0.38	-1.84	.0653
75,000–99,999	-0.90	0.34	-2.65	.0080
100,000 +	-1.69	0.34	-4.98	< .0001
	*Externalizing Behaviors*			
	B	SE	*t* value	*P*-value
Physical and Sexual Violence	0.32	0.10	3.28	.001
Parental Psychopathology	2.00	0.11	18.45	< .0001
Neighborhood Threat	0.97	0.11	8.56	< .0001
Prenatal Substance Exposure	1.14	0.12	9.67	< .0001
Scarcity	0.27	0.11	2.41	.0161
Household Dysfunction	1.07	0.12	16.98	< .0001
Age	-0.16	0.18	-0.86	.3909
Sex: Male	-1.45	0.18	-8.03	< .0001
Race and Ethnicity: White				
Black	-0.66	0.30	-2.17	.0299
Asian	-0.16	0.26	-0.62	.5353
Other	-2.09	0.64	-3.26	.0011
Hispanic	0.37	0.31	1.21	.2281
Family Income: 0–24,999				
25,000–49,999	-1.27	0.35	-3.63	.0003
50,000–74,999	-1.49	0.37	-4.05	< .0001
75,000–99,999	-1.44	0.33	-4.42	< .0001
100,000 +	-2.54	0.33	-7.82	< .0001
	*Total Behaviors*			
	B	SE	*t* value	*P*-value
Physical and Sexual Violence	0.46	0.11	4.36	.001
Parental Psychopathology	2.87	0.12	24.39	< .0001
Neighborhood Threat	1.15	0.12	9.31	< .0001
Prenatal Substance Exposure	1.01	0.13	7.9	< .0001
Scarcity	0.36	0.12	2.96	.0031
Household Dysfunction	1.90	0.13	14.32	< .0001
Age	-0.07	0.20	-0.35	.7253
Sex: Male	-2.01	0.20	-10.18	< .0001
Race and Ethnicity: White				
Black	-1.80	0.33	-5.47	< .0001
Asian	-0.08	0.28	-0.30	.7674
Other	-2.23	0.70	-3.20	.0014
Hispanic	0.50	0.34	1.48	.1390
Family Income: 0–24,999				
25,000–49,999	-0.62	0.38	-1.64	.1017
50,000–74,999	-1.12	0.40	-2.80	.0052
75,000–99,999	-1.31	0.36	-3.70	.0002
100,000 +	-2.22	0.35	-6.27	< .0001

**Table 5:** Binomial logistic regression of early life adversity and CBCL symptomology

	*Internalizing Behaviors*		
	OR	95% CI	*p*-value
Physical and Sexual Violence	1.09	1.05–1.14	< .0001
Parental Psychopathology	1.60	1.51–1.69	< .0001
Neighborhood Threat	1.20	1.13–1.27	< .0001
Prenatal Substance Exposure	1.07	1.01–1.12	.0197
Scarcity	1.06	1.01–1.12	.0243
Household Dysfunction	1.25	1.17–1.33	< .0001
Age	1.11	1.00–1.23	.0461
Sex: Male	0.59	0.53–0.65	< .0001
Race and Ethnicity: White			
Black	0.60	0.50–0.71	< .0001
Asian	1.13	0.99–1.30	.0758
Other	1.05	0.69–1.54	.8148
Hispanic	0.96	0.81–1.13	.6375
Family Income: 0–24,999			
25,000–49,999	0.90	0.75–1.08	.2518
50,000–74,999	0.84	0.69–1.02	.078
75,000–99,999	0.82	0.69–0.98	.0287
100,000 +	0.67	0.56–0.80	< .0001
	*Externalizing Behaviors*		
	OR	95% CI	*p*-value
Physical and Sexual Violence	1.05	0.99–1.10	0.046
Parental Psychopathology	1.30	1.52–1.75	< .0001
Neighborhood Threat	1.18	1.11–1.26	< .0001
Prenatal Substance Exposure	1.19	1.13–1.27	< .0001
Scarcity	1.05	0.98–1.12	.1205
Household Dysfunction	1.54	1.43–1.66	< .0001
Age	1.01	0.89–1.14	.9038
Sex: Male	0.58	0.51–0.66	< .0001
Race and Ethnicity: White			
Black	1.17	0.96–1.42	.1121
Asian	0.94	0.78–1.12	.5073
Other	0.43	0.17–0.89	.0418
Hispanic	1.32	1.08–1.61	.0052
Family Income: 0–24,999			
25,000–49,999	0.69	0.56–0.85	.0004
50,000–74,999	0.62	0.49–0.78	< .0001
75,000–99,999	0.66	0.54–0.81	< .0001
100,000 +	0.51	0.42–0.63	< .0001
	*Total Behaviors*		
	OR	95% CI	*p*-value
Physical and Sexual Violence	1.08	1.03–1.13	.0004
Parental Psychopathology	1.74	1.63–1.87	< .0001
Neighborhood Threat	1.20	1.12–1.27	< .0001
Prenatal Substance Exposure	1.17	1.10–1.23	< .0001
Scarcity	1.07	1.01–1.14	.0176
Household Dysfunction	1.51	1.41–1.62	< .0001
Age	1.01	0.90–1.14	.8333
Sex: Male	0.69	0.61–0.78	< .0001
Race and Ethnicity: White			
Black	0.94	0.78–1.14	.5313
Asian	1.03	0.87–1.21	.7305
Other	0.73	0.38–1.27	.3002
Hispanic	1.21	1.01–1.46	.0384
Family Income: 0–24,999			
25,000–49,999	0.79	0.65–0.96	.0158
50,000–74,999	0.63	0.51–0.79	< .0001
75,000–99,999	0.75	0.62–0.90	.0025
100,000 +	0.54	0.44–0.65	< .0001

*OR* odds ratio, *CI* confidence interval, *CBCL* symptomology was binarized such that (0 = normal functioning CBCL score of under 60; 1 = clinically-significant CBCL score of 60 and above)

**Table 6:** Relationship between early life adversity^a^ and CBCL symptomology

	*Internalizing Problematic Behaviors*			
*Adversity Type*	B	SE	*t* value	*p*-value
Physical and Sexual Violence	0.66	0.1	6.33	< .0001
Parental Psychopathology	2.83	0.11	25.62	< .0001
Neighborhood Threat	1.36	0.12	11.33	< .0001
Prenatal Substance Exposure	1.12	0.12	9.01	< .0001
Scarcity	0.22	0.12	1.81	.071
Household Dysfunction	1.44	0.13	11.12	< .0001
Cumulative Adversity	1.03	0.05	19.71	< .0001
	*Externalizing Problematic Behaviors*			
*Adversity Type*	B	SE	*t* value	*p*-value
Physical and Sexual Violence	0.55	0.1	5.43	< .0001
Parental Psychopathology	2.52	0.11	23.43	< .0001
Neighborhood Threat	1.49	0.12	12.79	< .0001
Prenatal Substance Exposure	1.78	0.12	14.83	< .0001
Scarcity	0.17	0.12	1.47	.141
Household Dysfunction	2.52	0.12	20.28	< .0001
Cumulative Adversity	1.17	0.05	23.29	< .0001
	*Total Problematic Behaviors*			
*Adversity Type*	B	SE	*t* value	*p*-value
Physical and Sexual Violence	0.74	0.11	6.64	< .0001
Parental Psychopathology	3.38	0.12	29.00	< .0001
Neighborhood Threat	1.74	0.13	13.61	< .0001
Prenatal Substance Exposure	1.85	0.13	14.06	< .0001
Scarcity	0.25	0.13	1.95	.052
Household Dysfunction	2.48	0.14	18.17	< .0001
Cumulative Adversity	1.36	0.05	24.85	< .0001

^a^Only one form of adversity exposure per model

## Discussion

### Overview

This study is, to our knowledge, the largest retrospective source of ELA derived from a population-based study of youth development. An EFA yielded a 6-factor solution corresponding to distinct domains of ELA, including: 1) physical and sexual violence; 2) parental psychopathology; 3) neighborhood threat; 4) prenatal substance exposure; 5) scarcity; and 6) household dysfunction. Our findings reveal that ELA prevalence among 9-and 10-year-old youth is largely driven by the incidence of parental psychopathology. The lifetime prevalence of any adult psychiatric disorder per DSM-IV diagnostic criteria has been estimated at 46.4% [24]. Our sample’s greater proportion (81.4%) may in part be attributable to a different measure being used to capture psychopathology and that the measure was not just completed by the youth’s biological parent but by the primary caregiver, which was not always the same. Therefore, parental psychopathology as an exposure may reflect both genetic and behavioral influences on our clinical outcomes and does not always equate with behavioral exposure in the instances where youth do not have contact with their biological parent(s) at baseline (n (%) = 443, 3.8%). Lastly, biological parental psychopathology reported by the caregiver is not equivalent to a clinical diagnosis.

Sociodemographic characteristics significantly differed between youth with adversity exposure and controls, specifically, sex, race/ethnicity of youth, primary caregiver’s education and family income. These findings are supported by previous research showing a higher incidence of ELA among racial and ethnic minorities, and among individuals identifying with low SES [4], the latter also associated with an increased risk for mental and physical health problems [25]. Adversity exposure was significantly associated with greater problematic behaviors, specifically, parental psychopathology, household dysfunction and neighborhood threat.

### Exploratory factor analysis

A 6-factor solution corresponding to 6 domains of ELA were derived from an EFA performed on 47 variables both youth and caregiver-reported across 14 measures. Seventeen variables were not included in the final EFA due to sparse endorsement of the variables which can in part be explained by the sensitive self-identifying nature of the questions, which were primarily caregiver-reported, as well as narrow time constraints referenced in the question, i.e., within the past 6 months. While the 6 domains of ELA are similar to the original ACEs, our domains differ in two prominent areas: incarceration of household member and neighborhood threat. We hypothesized that adversity domains derived from the EFA would overall align with and complement the domains established by the CDC-Kaiser ACE’s Study given that the original categorizations of exposure were broad yet discrete in nature. At baseline, the ABCD Study did not capture information on youth, caregiver, or household member incarceration. Given that one in three Americans will have an encounter with the criminal justice system, with racial and ethnic minorities carrying a significantly greater risk [26], capturing incidences of arrest, detainment, juvenile confinement, and adult incarceration are necessary to comprehensively catalog exposures that impact youth development. Not only does incarceration of a caregiver or family member constitute the removal of a source of support, a youth’s direct involvement with the justice system is associated with significant disadvantages (e.g., educational, economic, social, emotional, general health and wellbeing) throughout the lifespan [27]. Our study was, however, able to capture both youth and caregiver reported neighborhood threat. National survey data indicate that adolescent exposure to community violence is on par with adversity exposure within the home [28]. Irrespective of direct harm, community violence exposure constitutes a pervasive threat that accelerates biological aging and contributes to detrimental quality of life outcomes [29]. Despite not being captured in the original ACEs Study, more recent studies examining ELA are including measures of neighborhood or community threat and or violence [30]. Our findings support the literature detailing the increased incidence of problematic behaviors following neighborhood threat and community violence exposure [31]. Lastly, our EFA resulted in the combination of physical and sexual violence exposure into one domain versus two discrete categories of exposure. This may in part be explained by the minimal endorsement of these exposure types as well as that the same questionnaire (i.e., Kiddie Schedule for Affective Disorders and Schizophrenia (KSADS-5)) was used to measure physical and sexual violence exposure.

### Relationship between adversity and behavioral outcomes

Our findings that youth with ELA endorsed more internalizing, externalizing, and total problematic behaviors, which is associated with psychopathology risk, is supported in the literature [32]. Unsurprisingly, half of all childhood-onset and about one-third of adolescent-onset psychiatric disorders are associated with early life adversity exposure [33]. Our findings that parental psychopathology, household dysfunction, and neighborhood threat carried the greatest influence on problematic behaviors among 9-and 10-year-olds in our sample suggest identifying sources of resiliency that may combat these specific forms of exposure. For example, resources within the school and community, such as school-based programs, athletic associations, and peer mentorships, may act as sources of support for youth who are experiencing adversity within the home and immediate environment.

Youth with higher factor scores across the following domains had more internalizing problems: physical and sexual violence; parental psychopathology; and scarcity. Conversely, individuals with higher factor scores across the following domains had higher externalizing problems: neighborhood threat; prenatal substance exposure; and household dysfunction. While ELA exposure does not typically occur in insolation [8], these associations suggest possible mechanistic differences in type-specific ELA’s impact on associated behaviors. The mechanistic differences may be attributable to an individual’s neurodevelopmental stage during exposure and or to the neurodevelopmental subtilties in how different forms of ELA are processed in a region-specific manner. Understanding the nuanced relationship between subtypes of ELA and different problematic behaviors may aid in the earlier identification of ELA exposure and targeted interventional efforts, particularly for those that may be less physically-apparent (e.g., parental psychopathology).

### Implications of findings

Our findings spotlight the need to develop data-driven approaches to the categorization of ELA, highlighting the need to examine nuances of exposure, e.g., type, age of onset, frequency, duration, and relationship with the perpetrator. The youth in our sample endorsed discrete forms of ELA, the incidence of which significantly differed by sex, race, ethnicity and other sociodemographic characteristics. Additionally, different forms of ELA were associated with specific problematic behaviors. The use of broad domains, such as abuse and neglect [13]; active and passive adversity [14]; and threat and deprivation [15] in place of type-specific ELA in an attempt to categorize exposures and outcomes fails to account for the duality of some forms of adversity and the routine co-occurrence of exposures. For example, household dysfunction or family conflict could include both active and passive adversity exposure if the youth witnesses exposure but is also the direct recipient of. Neighborhood threat often co-occurs with deprivation, specifically, a greater prevalence of violence exposure within low SES communities [34], as well as food insecurity and social deprivation [35]. Despite the endorsement of neighborhood threat in our sample, exposure is not routinely examined and is at times even combined with low SES. Metrics of cumulative adversity and type-specific ELA should both be reported given the heterogeneity in sociodemographic associations and behavioral implications of ELA exposure; the utilization of broad categories and domains may inadvertently obscure pertinent associations and homogenize findings. A systematic data-driven approach to measure and categorize ELA in youth could aid not only in establishing a consistent manner with which to define and measure ELA, but could improve study reproducibility, and elucidate nuances in associated outcomes (e.g., behavioral and physiological) to improve evidence-based treatments and interventions. We advocate for the nuanced categorization of type-specific ELA as well as the inclusion of neighborhood threat as a form of exposure.

### Data-driven approaches to adversity categorization

To foster a systematic data-driven approach to measure and categorize ELA, we suggest the utilization of large publicly-available epidemiological datasets, including, the ABCD Study, Behavioral Risk Factor Surveillance System (BRFSS) surveys, and the National Longitudinal Study of Adolescent to Adult Health (Add Health). We recommend the utilization of rigorous yet generalizable statistical approaches, such as linear and logistic regression analyses with train and test models, in lieu of more dataset-specific methodologies, such as structural equation modeling [36–38]. While the modeling of complex patterns of variables and relationships is afforded by structural equation modeling, the analytical steps necessary to generate such a model result in findings that well-reflect the specific dataset, and thus, limit the generalizability.

## Limitations

The presence of early life adversity exposure captured in this study represents one time point (i.e., baseline) and may not be evident of chronic exposure. Additionally, the factor analysis is limited to the types of ELA exposure captured in the study. For example, household member incarceration and other forms of trauma, such as exposure to natural disasters, are not included. Several of the questions used to assess adversity exposure do not come from validated instruments. In instances where the caregiver may be unaware of exposure or may be associated either directly or indirectly with its perpetuation, the findings may not accurately reflect exposure. Given that most of the adversity exposure questions were answered by the caregivers, we hypothesize future EFAs of adversity exposure utilizing data from the ABCD Study to account for a greater proportion of variation in the data once youth self-report all adversity exposure. Thus, the proportion of variation explained by our six domains of ELA (i.e., 22.4%) is likely an underrepresentation of the true exposure and highlights the importance of developing questionnaires to capture ELA in youth, either utilizing more indirect questioning for caregivers, or employing developmentally considerate questions completed by youth. Of note, the utility of a factor model is not best captured by percent variance explained but by the performance of the model’s fit indices, e.g., RMSEA, TFI and CFI. As youth age, the ABCD study will continue to obtain information regarding adversity exposure, allowing variables that are captured at discrete timepoints to be related to one another. Despite the strengths of population-based studies, a limitation of this and other studies not specifically designed to investigate ELA exposure are the less detailed and nuanced questions used to assess exposure.

## Conclusions

Given the prevalence of ELA exposure, the acute and long-term implications of exposure across a variety of domains, as well as the limited replicability and inconsistent findings, we recommend a systematic data-driven approach to measure and categorize adversity exposure in youth. Data-driven approaches to defining and categorizing ELA are likely to enhance our understanding of the physiological mechanisms associated with outcomes risk and resiliency aptitude following exposure. To do so, we suggest the incorporation of more versus less data by capturing the nuances of exposure (e.g., type, age of onset, frequency, duration) and utilizing publicly available longitudinal datasets. Broad categorizations, including abuse and neglect and threat and deprivation, fail to account for the routine co-occurrence of exposures and the duality of some forms of adversity. The use of a data-driven, standardized methodology to define and measure ELA is a crucial step to lessening barriers to evidence-based treatments and interventions for youth.

## Supplementary Information

**Additional file 1.**

## Data Availability

The datasets generated and analyzed during the current study are available in The ABCD Data Repository, https://nda.nih.gov/abcd/.

## Abbreviations

ELA: Early life adversity
SES: Socioeconomic status
ACEs: Adverse childhood experiences
MACE: Maltreatment and Abuse Chronology of Exposure
ABCD: Adolescent Brain Cognitive Development
EFA: Exploratory factor analysis
CBCL: Child Behavior Checklist
RMSEA: Root mean square error of approximation
TFI: Tucker-Lewis index
CFI: Comparative fit index
DSM-IV: Diagnostic and Statistical Manual of Mental Disorders, fourth edition
BRFSS: Behavioral Risk Factor Surveillance System
Add Health: National Longitudinal Study of Adolescent to Adult Health
