# Supplementary material for: A data-driven approach to categorizing early life adversity exposure in the ABCD Study
Source: BMC Med Res Methodol. 2023 Jul 7;23:164. doi: 10.1186/s12874-023-01983-9 (PMC10327383; doi:10.1186/s12874-023-01983-9)
Supplement: Supplementary file 1 — Additional file 1. [file 12874_2023_1983_MOESM1_ESM.docx]

SUPPLEMENT

Table S1: Questions utilized in the final exploratory factor analysis, organized by factor domains. Parent-reported unless otherwise indicated.

**Physical and Sexual Violence**

1. Shot, stabbed, or beaten brutally by a non-family member? From: Kiddie Schedule for Affective Disorders and Schizophrenia (KSADS-5)
2. Shot, stabbed, or beaten brutally by a grown up in the home? From: Kiddie Schedule for Affective Disorders and Schizophrenia (KSADS-5)
3. Beaten to the point of having bruises by a grown up in the home? From: Kiddie Schedule for Affective Disorders and Schizophrenia (KSADS-5)
4. A grown up in the home touched your child in his or her privates, had your child touch their privates, or did other sexual things to your child? From: Kiddie Schedule for Affective Disorders and Schizophrenia (KSADS-5)
5. An adult outside your family touched your child in his or her privates, had your child touch their privates, or did other sexual things to your child? From: Kiddie Schedule for Affective Disorders and Schizophrenia (KSADS-5)
6. A peer forced your child to do something sexually? From: Kiddie Schedule for Affective Disorders and Schizophrenia (KSADS-5)
7. Witnessed someone shot or stabbed in the community From: Kiddie Schedule for Affective Disorders and Schizophrenia (KSADS-5)
8. A non-family member threatened to kill your child? From: Kiddie Schedule for Affective Disorders and Schizophrenia (KSADS-5)
9. A family member threatened to kill your child? From: Kiddie Schedule for Affective Disorders and Schizophrenia (KSADS-5)

**Parental Psychopathology**

1. Has the child’s biological parents ever had any problems due to alcohol, such as: Marital separation or divorce; Laid off or fired from work; Arrests or DUIs; Alcohol harmed their health; In an alcohol treatment program; Suspended or expelled from school 2 or more times; Isolated self from family, caused arguments or were drunk a lot. From: ABCD Family History Assessment
2. Has the child’s biological parents ever had any problems due to drugs, such as: Marital separation or divorce; Laid off or fired from work; Arrests or DUIs; Drugs harmed their health; In a drug treatment program; Suspended or expelled from school 2 or more times; Isolated self from family, caused arguments or were high a lot. From: ABCD Family History Assessment
3. Has the child’s biological parents ever suffered from depression, that is, have they felt so low for a period of at least two weeks that they hardly ate or slept or couldn't work or do whatever they usually do? From: ABCD Family History Assessment
4. Has the child’s biological parents ever had a period of time when others were concerned because they suddenly became more active day and night and seemed not to need any sleep and talked much more than usual for them? From: ABCD Family History Assessment
5. Has the child’s biological parents ever had a period lasting six months when they saw visions or heard voices or thought people were spying on them or plotting against them? From: ABCD Family History Assessment
6. Has the child’s biological parents ever been to a doctor or a counselor about any emotional or mental problems, or problems with alcohol or drugs? From: ABCD Family History Assessment
7. Has the child’s biological parents ever been hospitalized because of emotional or mental problems, or drug or alcohol problems? From: ABCD Family History Assessment
8. Has the child’s biological parents ever attempted or committed suicide? From: ABCD Family History Assessment

**Neighborhood Threat**

1. My neighborhood is safe from crime. From: ABCD Parent Neighborhood Safety/Crime Survey modified from PhenX (NSC)
2. Violence is not a problem in my neighborhood. From: ABCD Parent Neighborhood Safety/Crime Survey modified from PhenX (NSC)

**Prenatal Substance Exposure**

Once you knew you were pregnant, were you using any of the following:

1. tobacco
2. alcohol
3. marijuana
4. cocaine/crack
5. heroin/morphine
6. OxyContin

All From: ABCD Developmental History Questionnaire

**Scarcity**

In the past 12 months, has there been a time when you and your immediate family experienced any of the following:

1. Needed food but couldn’t afford to buy it or couldn’t afford to go out to get it? From: ABCD Parent Demographics Survey
2. Had services turned off by the gas or electric company, or the oil company wouldn’t deliver oil because payments were not made? From: ABCD Parent Demographics Survey

**Household Dysfunction**

1. Family members sometimes hit each other. (Youth-reported) From: ABCD Youth Family Environment Scale-Family Conflict Subscale modified from PhenX (FES)
2. We fight a lot in our family. (Youth-reported) From: ABCD Youth Family Environment Scale-Family Conflict Subscale modified from PhenX (FES)
3. Family members often criticize each other. (Youth-reported) From: ABCD Youth Family Environment Scale-Family Conflict Subscale modified from PhenX (FES)

Table S2: Questions not included in the final exploratory factor analysis due to factor loading below 0.4. Parent-reported unless otherwise indicated.

1. Has he/she ever been to a doctor, a nurse, nurse practitioner, the emergency room or a clinic because any of these things happened: wound from knife or any other weapon? From: ABCD Parental Medical History Questionnaire
2. Has he/she ever been to a doctor, a nurse, nurse practitioner, the emergency room or a clinic because any of these things happened: gunshot wound? From: ABCD Parental Medical History Questionnaire
3. I scream or yell a lot. *Question is in reference to the past 6 months.* From: ABCD Parent Adult Self Report
4. My [caregiver] is a person who believes in showing his/her love for me. (Youth-reported) From: ABCD Children’s Report of Parental Behavior Inventory (CRPBI)
5. My [caregiver] is a person who is easy to talk to. (Youth-reported) From: ABCD Children’s Report of Parental Behavior Inventory (CRPBI)
6. My [caregiver] is a person who is able to make me feel better when I am upset. (Youth-reported) From: ABCD Children’s Report of Parental Behavior Inventory (CRPBI)
7. How often do your parents/guardians know where you are? (Youth-reported) From: ABCD Parental Monitoring Survey
8. I meet my responsibilities to my family. From: ABCD Parent Adult Self Report
9. In the past 12 months, has there been a time when you and your immediate family experienced any of the following: Were evicted from your home for not paying the rent or mortgage? From: ABCD Parent Demographics Survey
10. I drink too much alcohol or get drunk a lot. From: ABCD Parent Adult Self Report
11. I use drugs (other than alcohol, nicotine) for non-medical purposes. From: ABCD Parent Adult Self Report
12. Once you knew you were pregnant, were you using any of the following: any other drugs? From: ABCD Developmental History Questionnaire
13. I deliberately try to hurt or kill myself. From: ABCD Parent Adult Self Report
14. I feel safe at my school. (Youth-reported) From: ABCD School Risk and Protective Factors Survey
15. My neighborhood is safe from crime. (Youth-reported) From: ABCD Youth Neighborhood Safety/Crime Survey modified from PhenX (NSC)
16. Does your child have any problems with bullying at school or in your neighborhood? From: Kiddie Schedule for Affective Disorders and Schizophrenia (KSADS-5)
17. Witness the grownups in the home push, shove or hit one another? From: Kiddie Schedule for Affective Disorders and Schizophrenia (KSADS-5)
